# An outbreak of *bla*_KPC−4_- and *bla*_VIM−1_-producing *Klebsiella pneumoniae* and *Klebsiella variicola* at a single hospital in South Korea

**DOI:** 10.1186/s13756-024-01478-2

**Published:** 2024-10-11

**Authors:** Jiyon Chu, Jaeki Choi, Seul Ki Ji, Chulmin Park, Seung-Hyun Jung, Sun Hee Park, Dong-Gun Lee

**Affiliations:** 1 Department of Medical Sciences, College of Medicine, The Catholic University of Korea, Seoul, Republic of Korea; 2 Division of Infectious Diseases, Department of Internal Medicine, College of Medicine, The Catholic University of Korea, Seoul, Republic of Korea; 3 Vaccine Bio Research Institute, College of Medicine, The Catholic University of Korea, Seoul, Republic of Korea; 4 Infection Prevention and Control Unit, Daejeon St. Mary’s Hospital, The Catholic University of Korea, Daejeon, Republic of Korea; 5 Integrated Research Center for Genome Polymorphism, College of Medicine, The Catholic University of Korea, Seoul, Republic of Korea; 6 Department of Biochemistry, College of Medicine, The Catholic University of Korea, Seoul, Republic of Korea

**Keywords:** *Klebsiella pneumoniae*, *Klebsiella variicola*, *bla*_KPC−4_ and *bla*_VIM−1_, Colistin resistance, Whole-genome sequencing

## Abstract

**Background:**

The dissemination of *Klebsiella* spp. producing multiple carbapenemases has been increasingly recognized. Between July 2019 and August 2021, ten patients were found to carry *Klebsiella* spp. co-harboring *bla*_KPC−4_ and *bla*_VIM−1_ across multiple wards at a Korean hospital, and one isolate was recovered from a hand-washing sink, more than a year after the outbreak. This study aimed to investigate the outbreak and conduct a genomic study of these isolates.

**Methods:**

Whole-genome sequencing, including long-read sequencing, was performed to analyze plasmid structures and mobile genetic elements (MGEs). Bioinformatics analyses were performed to trace clonal transmission chains and horizontal gene transfer.

**Results:**

The findings suggested that the inter-ward spread of *Klebsiella* spp. seemed to be facilitated by healthcare worker contact or patient movement. Of the nine isolates collected (eight clinical and one environmental), seven (including the environmental isolate) were identified as *K. pneumoniae* (ST3680) and two were *K. variicola* (single-locus variant of ST5252). These isolates showed high genetic relatedness within their species and harbored the IncHI5B plasmid carrying both *bla*_KPC−4_ and *bla*_VIM−1_ (pKPCVIM.1). On this plasmid, *bla*_VIM−1_ was located in the Class 1 integron associated with IS*1326*::IS*1353* (In2), and Tn*4401b* carrying *bla*_KPC−4_ was inserted into IS*1326*::IS*1353*, creating a novel MGE construct (In2_*bla*_VIM−1_-Tn*4401b*_*bla*_KPC−4_).

**Conclusion:**

The hospital-wide spread of *bla*_KPC−4_ and *bla*_VIM−1_ was facilitated by clonal spread and horizontal plasmid transfer. The persistence of this strain in the hospital sink suggests a potential reservoir of the strain. Understanding the transmission mechanisms of persistent pathogens is important for improving infection control strategies in hospitals.

**Supplementary Information:**

The online version contains supplementary material available at 10.1186/s13756-024-01478-2.

## Background

Carbapenemase-producing Enterobacterales (CPE) pose a significant threat to global health. In South Korea, CPE cases have steadily increased after the first imported case in 2010 [1], with 21,695 cases reported in 2022 [2]. Three main types of carbapenemases exist: class A *Klebsiella pneumoniae* carbapenemase (KPC), class B metallo-β-lactamases (MBLs; predominantly imipenemase [IMP]-, verona integron-encoded [VIM]-, and New Delhi [NDM]-type enzymes), and class D oxacillinases (OXAs). These carbapenemases reside on various conjugative plasmids and spread horizontally through promiscuous plasmids and/or mobile genetic elements (MGEs), such as integrons and transposons, common in Enterobacterales. KPC dissemination is facilitated by the highly mobile Tn3-based transposon Tn4401, whereas VIM-1 spread is associated with the Class I integron, containing gene cassettes encoding antibiotic resistance genes (ARGs) [3].

A CPE outbreak occurred from July to September 2019 across multiple wards at Daejeon St. Mary’s Hospital, South Korea, despite the implementation of a CPE admission screening program since September 2018. In response, extensive contact tracing and an enhanced CPE screening program were implemented, continuing post-outbreak. By December 2020, 167 new patients positive for CPE were identified. The CPE isolates and carbapenemase genes exhibited diversity, with over 19 distinct species/carbapenemase gene combinations identified [4]. Notably, ten patients were colonized with *Klebsiella pneumoniae*, producing both *bla*_KPC−4_ and *bla*_VIM−1_ [4]. Additionally, one *K. pneumoniae* isolate co-producing these genes was detected in the environment more than a year after the outbreak ended. Although *K. pneumoniae* strains co-harboring other VIM-type MBLs and KPC-type carbapenemases have been occasionally reported [5–8], the co-existence of *bla*_KPC−4_ and *bla*_VIM−1_ is unprecedented in South Korea and globally.

In this study, we aimed to investigate the in-hospital transmission mechanism of *bla*_KPC−4_ and *bla*_VIM−1_ and the potential environmental role in the spread of isolates co-producing *bla*_KPC−4_ and *bla*_VIM−1_ using complete genome sequencing.

## Methods

### Hospital setting

Daejeon St. Mary’s Hospital, a 660-bed university-affiliated secondary care facility in Daejeon, South Korea, serves a population of 1.5 million people, with 24,300 annual admissions. The hospital wards predominantly comprise multi-occupancy rooms (95%) with shared bathrooms, along with single rooms with en-suite bathrooms (5%) and four specialized rooms for airborne infection isolation. The two intensive care units (ICUs) feature an open bay layout with two isolation rooms each. Approximately 22% of inpatients had malignancy, and the mean age of inpatients was 62.4 (standard deviation ± 16.9) years.

This hospital encountered sporadic CPE cases after the identification of the first case (*bla*_NDM_-positive *Escherichia coli*) in April 2017. In September 2018, a CPE screening program was initiated targeting previously colonized individuals, those admitted at other healthcare facilities within one month, and all patients in the ICU upon admission, with 59.3% compliance. However, an outbreak occurred from July to September 2019, prompting extensive contact tracing and the expansion of the CPE screening program using Xpert-Carba-R and cultures. Post-outbreak, the enhanced CPE screening continued, reaching 93.5% compliance [4]. Despite stable rates of new cases, sporadic in-hospital cases persisted, prompting the implementation of environmental surveillance cultures across all wards and ICUs from February to August 2021.

This study was approved by the Institutional Review Board of the Catholic University of Korea, Daejeon St. Mary’s Hospital (DC21ENSI0040). The requirement for informed consent was waived.

### Study population and microbiological analyses

Patients colonized/infected with *Klebsiella* spp. co-producing *bla*_KPC−4_ and *bla*_VIM−1_ during the outbreak period through August 2021 were identified, and isolates were collected. Clinical characteristics and risk factors were compared with age, sex, and time-matched controls not colonized with CPE. One environmental isolate was detected from a ward sink during environmental surveillance. Carbapenemase genes were subtyped using PCR and Sanger sequencing [9]. Initial species identification was performed using matrix-assisted laser desorption/ionization time-of-flight mass spectrometry (MALDI-TOFMS) (Bruker, Daltonics, Germany), confirmed by 16s rRNA sequencing. Antimicrobial susceptibilities were determined using a MicroScan WalkAway 96 Plus system and Neg Combo Panel Type 72 (Beckman Coulter, Brea, CA). The broth microdilution method was additionally performed to determine susceptibilities to imipenem, meropenem, ertapenem, and colistin according to the 2019 Clinical and Laboratory Standards Institute guidelines. Nine isolates (eight clinical and one environmental) were used for further genomic analysis.

### Whole-genome sequencing and *de novo* assembly

Nine isolates were subjected to short-read sequencing using the Illumina NovaSeq system (Illumina, San Diego, CA), and five were additionally subjected to long-read sequencing using the PacBio system (Pacific Biosciences, Menlo Park, CA) to obtain complete plasmid sequences. Sequencing reads from the Illumina and PacBio systems were assembled using SPAdes [10] and SMRT Portal (Pacific Biosciences), respectively. Assembled contigs were annotated using Prokka [11]. Raw sequencing reads were deposited in the Sequence Read Archive under accession number PRJNA1079714.

### Molecular typing

Species, multilocus sequence type, ARGs, virulence factors, and capsule/O-antigen biosynthesis locus type were determined using Kleborate [12]. ARGs were verified using the Comprehensive Antibiotic Resistance Database [13]. Mobilomes were identified using IntegronFinder [14] and MobileElementFinder [15]. Plasmids were classified according to replicon and MOB typing using PlasmidFinder [16], KpVR [17], and MOB-Suite [18]. The presence of IncHI5 plasmids was further confirmed using repHI5B on pKOX_R1 (GenBank accession: CP003684) through BLAST alignment (> 95% identity and 100% coverage). Horizontal plasmid transfer between *K. pneumoniae* and *K. variicola* was identified based on the empirical similarity threshold of < 15 single nucleotide polymorphisms (SNPs) per 100 Kb of plasmid sequence [19]. Plasmid sequence comparison, including isolates with only Illumina sequencing data, was performed using BLAST and visualized using Proksee [20]. Genomic structures surrounding *bla*_KPC−4_ and *bla*_VIM−1_ were visualized using the gggenes R-package. Core gene alignment was performed based on the annotated contigs, and a maximum likelihood phylogenetic tree was constructed based on core gene SNPs using RAxML [21]. Genetic relatedness of chromosomes and plasmids was assessed by calculating the average nucleotide identity of the core gene. Detailed methods for whole-genome sequencing (WGS) analyses, molecular typing, and phylogenetic analyses are provided in Additional file 1, and details of genome-based molecular typing are provided in Additional file 2: Table S1.

### Statistical analysis

Categorical and continuous variables were compared using Fisher’s exact test and Student’s *t*-test or Wilcoxon rank-sum test, respectively. Risk factors were assessed using conditional logistic regression models with odd ratios and 95% confidence intervals. To identify independent risk factors, a multivariate model with forward stepwise selection was performed for variables with *P* < 0.05 in the bivariate analyses. For all tests, *P* < 0.05 was considered statistically significant. All analyses were performed using Stata (version 18.0; StataCorp LP, College Station, TX).

## Results

### Description of cases

**Fig. 1:**
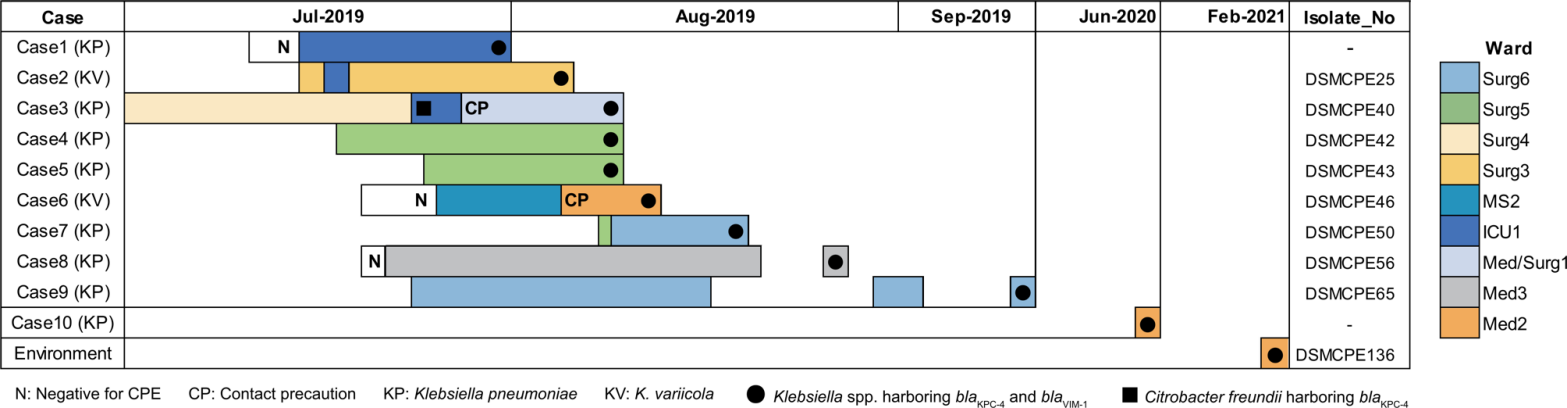
Patient admissions and movement in hospital until the date *Klebsiella* spp. co-producing *bla*_KPC−4_ and *bla*_VIM−1_ were detected during the outbreak (July to September 2019) and post-outbreak (October 2019 to February 2021)

Between July 2019 and August 2021, ten cases were identified through rectal swab screenings across six different wards and one ICU (Fig. 1). The first two cases (Cases 1 and 2) were detected through contact tracing of a patient (Case 3) with *bla*_KPC−4_-positive *Citrobacter freundii* in urine. This patient (Case 3) subsequently found colonized with *K. pneumoniae* co-harboring *bla*_KPC−4_ and *bla*_VIM−1_. Furthermore, contact tracing of patients colonized with *bla*_NDM−1_- or *bla*_KPC−2_-positive CPE revealed four cases (Cases 4–7). Admission screenings between August and September 2019 and June 2020 revealed three cases (Cases 8–10), all with recent admission to this hospital within the previous six months. Environmental surveillance in February 2021 revealed one isolate from a sink in the nurse station of the Medical-2 ward. Initially, all isolates were identified as *K. pneumoniae*; however, subsequent WGS identified two isolates (Case 2 and 6) as *K. variicola*. Demographics and clinical characteristics are summarized in Table 1.

**Table 1:** Demographics and characteristics of cases colonized with *bla*_KPC−4_ and *bla*_VIM−1_ producing *Klebsiella* spp. and comparisons with controls

Clinical parameters^*^	Cases^†^ (*N* = 10)	Controls^†^ (*N* = 40)	*P*-value	aOR^††^ (95% CI)	*P*-value
Age, years, mean (SD)	65.7 (12.8)	66.7 (12.3)	0.817		
Male, (%)	6 (60.0)	25 (62.5)	1.000		
CCI, mean (SD)	3.0 (4.0)	2.6 (2.5)	0.709		
Transfer from other HCFs	3 (30.0)	2 (5.0)	0.048	60.3 (0.8-4387.2)	0.061
Previous admission	5 (50.0)	13 (32.5)	0.463		
Antimicrobial therapy	10 (100.0)	27 (67.5)	0.046		
Procedures					
Bronchoscopy	0 (0.0)	3 (7.5)	1.000		
Cystoscopy	0 (0.0)	1 (2.5)	1.000		
Gastroenteroscopy	0 (0.0)	4 (10.0)	0.571		
Colonoscopy	0 (0.0)	2 (5.0)	1.000		
Cardiac intervention	2 (20.0)	5 (12.5)	0.616		
Radiology intervention	3 (30.0)	12 (30.0)	1.000		
Operation	6 (60.0)	16 (40.0)	0.302		
Central line	2 (20.0)	1 (2.5)	0.098		
Urinary catheter	5 (50.0)	9 (22.5)	0.118		
Drain (any site)	5 (50.0)	3 (7.5)	0.005	12.0 (0.8-174.2)	0.069
Physical therapy	1 (10.0)	2 (5.0)	0.496		
Locations					
Medical2	2 (20.0)	3 (7.5)	0.258		
Medical3	1 (10.0)	0 (0.0)	0.200		
Surgical3	1 (10.0)	1 (2.5)	0.363		
Surgical4	1 (10.0)	3 (7.5)	1.000		
Surgical5	3 (30.0)	2 (5.0)	0.048		
Surgical6	2 (20.0)	7 (17.5)	1.000		
Med/Surg2	1 (10.0)	4 (10.0)	1.000		
Med/Surg3	1 (10.0)	2 (5.0)	0.496		
ICU1	3 (30.0)	2 (5.0)	0.048		
Department					
Surgery	1 (10.0)	12 (30.0)	0.258		
Cardiology	2 (20.0)	16 (40.0)	0.295		
Gastroenterology	2 (20.0)	8 (20.0)	1.000		
Oncology	1 (10.0)	5 (12.5)	1.000		
Orthopedic surgery	6 (60.0)	5 (12.5)	0.004	15.9 (1.2-212.2)	0.036

^*^Values are no. (%) except as indicated

^†^Cases are patients colonized with *Klebsiella* spp. co-producing *bla*_KPC−4_ and *bla*_VIM−1_, while controls are patients who were not colonized with carbapenemase-producing Enterobacterales

^††^Adjusted odds ratios (aOR) of variables, such as antimicrobial therapy and specific locations (Surgical5, ICU1), which were excluded from the final model during the stepwise selection process, are not displayed

aOR: adjusted odds ratio, CCI: Charlson Comorbidity Index, CI: confidence interval, HCF: healthcare facility, ICU: Intensive Care Unit, SD: standard deviation

**Fig. 2:**
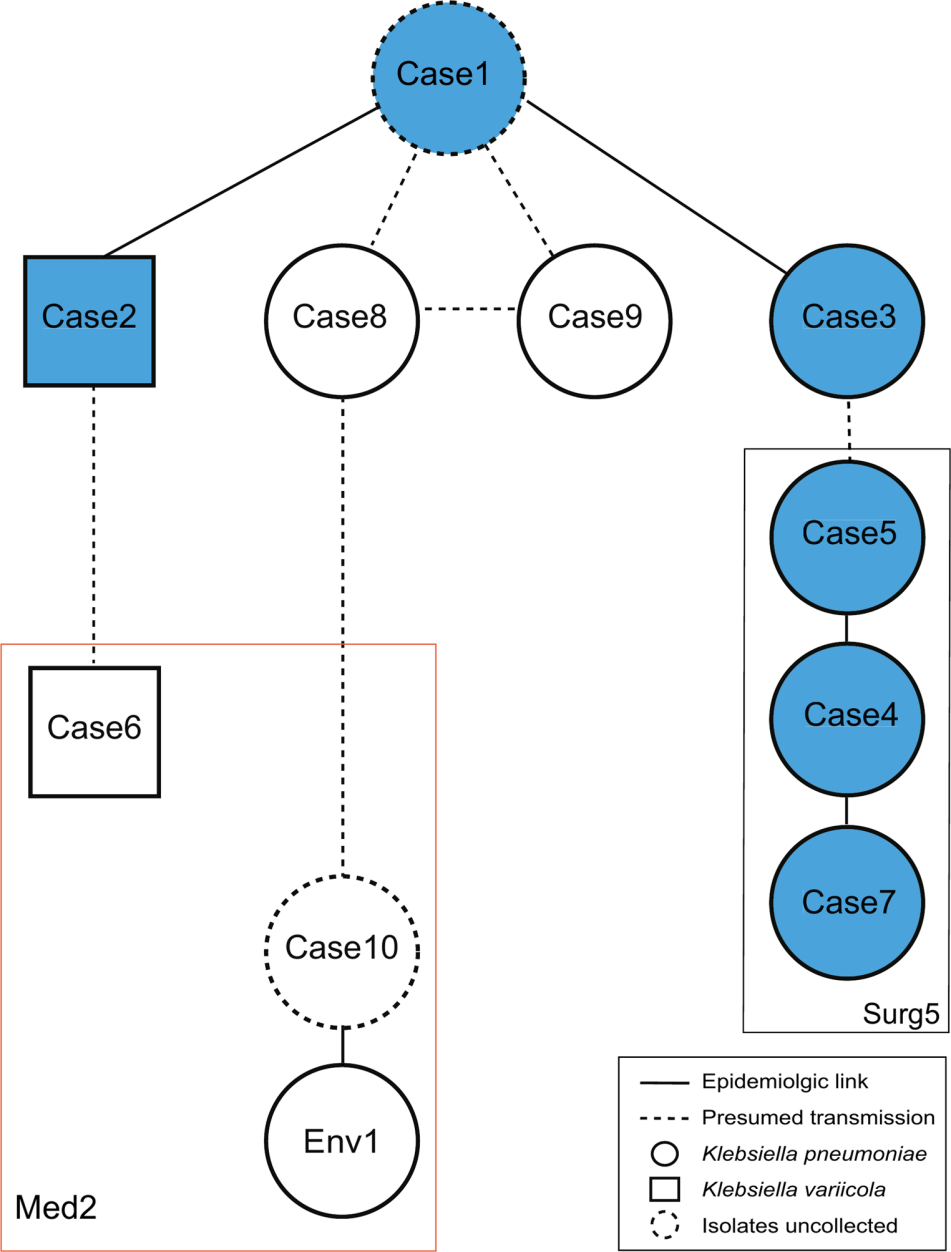
Potential transmission routes of pKPCVIM.1 plasmid between *Klebsiella pneumoniae* and *Klebsiella variicola* based on the epidemiologic links and genetic relatedness. Solid lines indicate known epidemiologic links while dotted lines indicate presumed transmission links. Squares and circles symbolize *K. variicola* and *K. pneumoniae*, respectively, with dotted circles indicating cases where isolates were not collected. Blue-filled circles and squares indicate cases associated with the orthopedic surgery department

Despite being detected in various locations, six cases were associated with the orthopedic surgery department, with five patients under orthopedic surgery care and one ICU patient under hepatology care consulting orthopedics for fasciotomy (Table 1; Fig. 2; Additional file 1: Fig. S1). These cases shared healthcare personnel, including two nurses, one intern, and four residents, indicating potential intra-departmental transmission. The other four cases lacked an epidemiological link to orthopedics, with no identified common exposure source. Between the two *K. variicola* cases (Cases 2 and 6), no direct link was identified. After ten months of no detection, a new case (Case 10) emerged in the Medical-2 ward, where an environmental isolate was identified in a sink at the nurse station nine months after Case 10 was detected. Patients who were monitored using rectal swabs (*n* = 8) tested negative for CPE within a median of 18 days (7–293 days) from the initial detection without further clinical infections.

### Antimicrobial susceptibilities and genomic characteristics

All isolates carrying *bla*_KPC−4_ and *bla*_VIM−1_ were highly resistant to meropenem, imipenem, and ertapenem (Table 2), as well as to aminoglycosides, and 67% of cases to fluoroquinolones. Seven isolates (77.8%) were resistant to colistin, harboring a truncating mutation in the *mgrB* gene. However, all isolates were susceptible to fosfomycin, trimethoprim/sulfamethoxazole, and tigecycline (Additional file 1: Table S2).

**Table 2:** Antimicrobial susceptibility profile of *Klebsiella* spp. co-harboring *bla*_KPC−4_ and *bla*_VIM−1_

Isolate ID	Species	Antimicrobial susceptibility test (MIC, µg/mL)								Related ARGs other than bla_KPC−4_ and bla_VIM−1_^*^
		MER	IPM	ERT	COL	GEN	TOB	CIP	LVX	
DSMCPE25	*K. variicola*	64	32	64	2	8	> 8	<=1	<=2	*aac(6’)-Ib9, aadA1, aadA2, qnrA1*
DSMCPE46	*K. variicola*	64	32	32	4	8	> 8	<=1	<=2	*aac(6’)-Ib9, aadA1, aadA2, qnrA1*
DSMCPE40	*K. pneumoniae*	8	16	8	64	8	> 8	2	2	*mgrB, aac(6’)-Ib9, aadA1, qnrS1*
DSMCPE42	*K. pneumoniae*	32	16	16	32	8	> 8	> 2	2	*mgrB, aac(6’)-Ib9, aadA1, qnrS1*
DSMCPE43	*K. pneumoniae*	32	16	16	64	8	> 8	> 2	4	*mgrB, aac(6’)-Ib9, aadA1, qnrS1*
DSMCPE50	*K. pneumoniae*	32	16	16	0.5	8	> 8	<=0.5	<=1	*aac(6’)-Ib9, aadA1, qnrS1*
DSMCPE56	*K. pneumoniae*	32	32	16	16	8	> 8	> 2	2	*mgrB, aac(6’)-Ib9, aadA1, qnrS1*
DSMCPE65	*K. pneumoniae*	64	32	32	32	8	> 8	> 2	4	*mgrB, aac(6’)-Ib9, aadA1, qnrS1*
DSMCPE136	*K. pneumoniae*	64	64	32	128	8	> 8	> 2	4	*mgrB, aac(6’)-Ib9, aadA1, qnrS1*

*Regarding *mgrB* gene, only isolates in which the truncated mutation was detected were listed

MIC: minimum inhibitory concentration, MER: meropenem, IPM: imipenem, ERT: ertapenem. COL: colistin, GEN: gentamicin, TOB: tobramycin, CIP: ciprofloxacin, LVX: levofloxacin

**Fig. 3:**
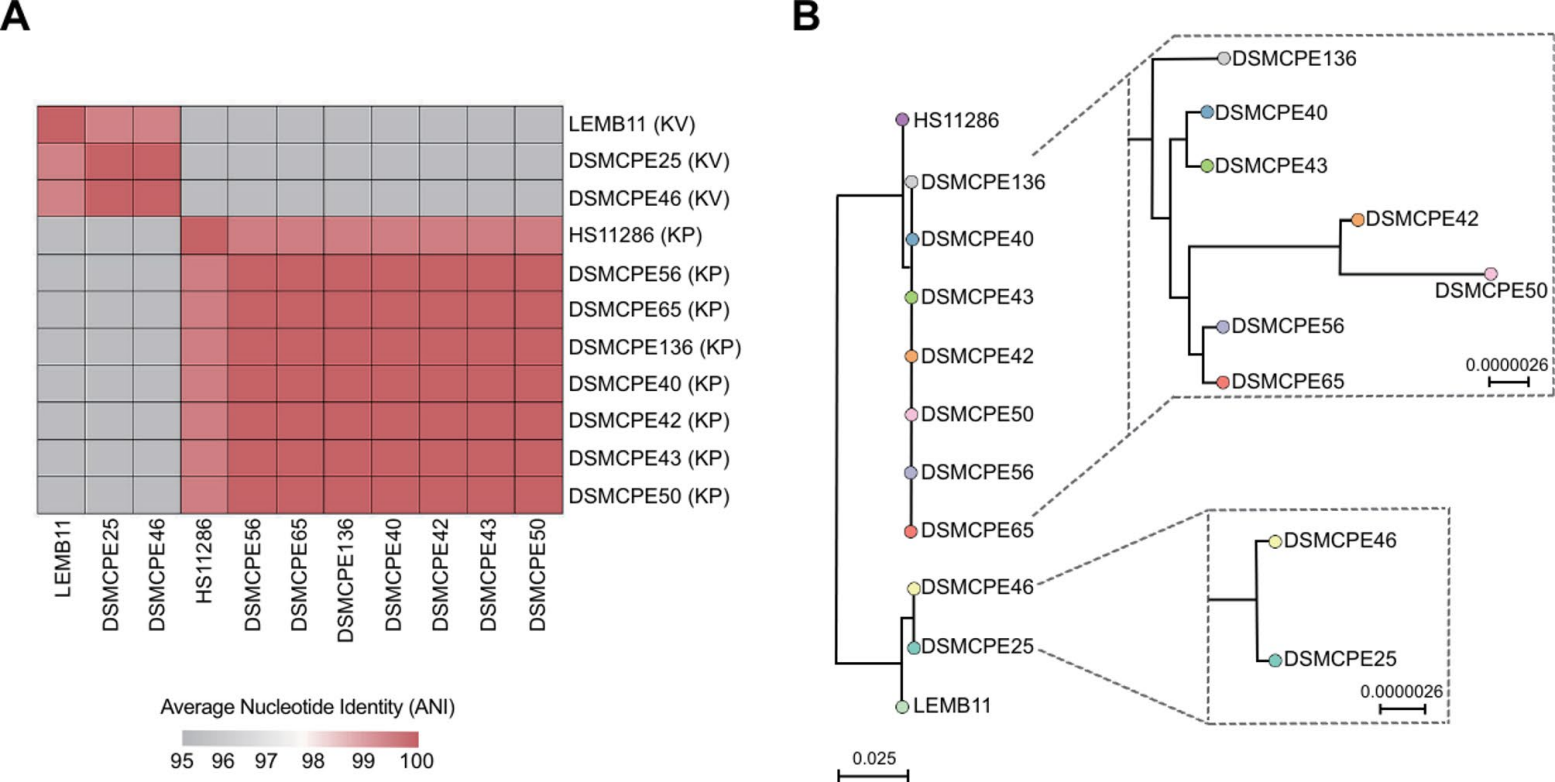
Genetic relationship between *Klebsiella* spp. isolates. (**A**) SNP difference matrix of *Klebsiella* spp. co-harboring *bla*_KPC−4_ and *bla*_VIM−1_. The similarity of the isolates was assessed based on the chromosomal average nucleotide identity between the core genes of the isolates, with < 25 differential SNPs considered identical within the species. (**B**) Maximum likelihood phylogenetic tree of *Klebsiella* spp. isolates. The *K. pneumoniae subsp. pneumoniae* HS11286 was used as an outgroup to root the tree. The scale represents the number of SNPs per variable site. KP: *K. pneumoniae*, KV: *K. variicola*

SNP analysis revealed that all *K. pneumoniae* isolates belonged to ST3680 with high genetic relatedness (Fig. 3). The environmental isolates also shared genetic similarities with clinical isolates detected 2.5 years earlier. The two *K. variicola* isolates, identified as single-locus variants of ST5252, were genetically related but significantly divergent from the reference genome (Fig. 3).

### Plasmid analysis

**Fig. 4:**
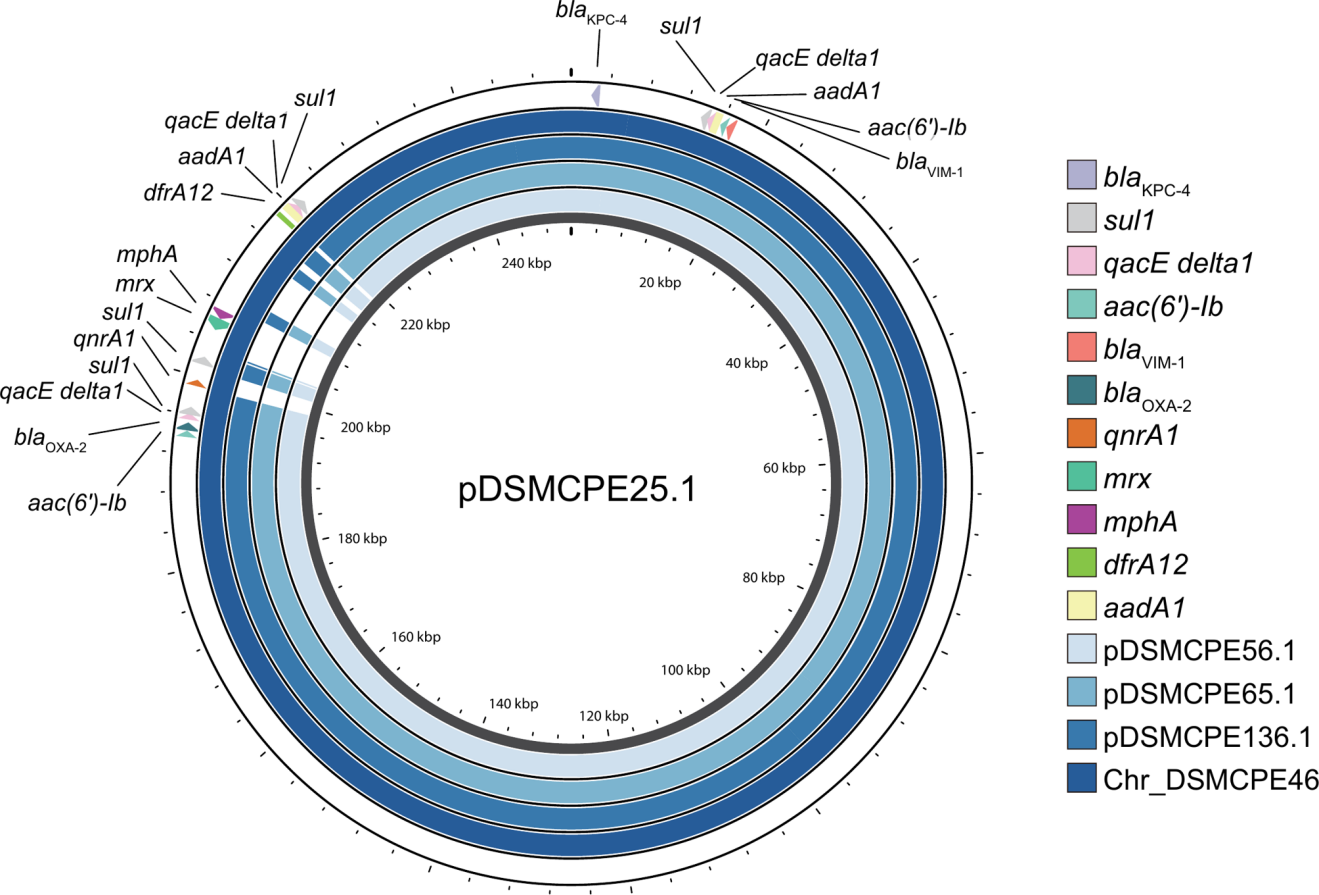
Comparison of plasmids carrying *bla*_KPC−4_, *bla*_VIM−1_, and *bla*_OXA−2_ genes. BLAST alignment of three plasmids from *K. pneumoniae* isolates (DSMCPE56, DSMCPE65, and DSMCPE136) and one chromosome from *K. variicola* isolate (DSMCPE46) for which complete genome sequencing data was acquired. The circular map was generated using Proksee, and a 252-Kb sized plasmid recovered from DSMCPE25 (pDSMCPE25.1) was used as the reference sequence

Complete genome sequences of five isolates (Cases 2 [DSMCEP25], 6 [DSMCPE46], 8 [DSMCPE56], and 9 [DSMCPE65] and the environmental isolate [DSMCEP136]) revealed 1–3 plasmids per isolate. Notably, two types of plasmids carrying *bla*_KPC−4_ and *bla*_VIM−1_ were identified: one with *bla*_OXA−2_ (pKPCVIM.1) and one without (pKPCVIM.2). pKPCVIM.1, classified as IncHI5B and MOBH, was identified in four isolates (three *K. pneumoniae* and one *K. variicola*), whereas pKPCVIM.2, classified as IncR and MOBF, was detected in two *K. variicola* isolates (Additional file 1: Table S3). Although the pKPCVIM.1 plasmid size of *K. variicola* (pDSMCPE25.1 from Case 1, 252 Kb) was larger than that of *K. pneumoniae* (pDSMCPE56.1 and pDSMCPE65.1, 227 Kb; pDSMCPE136, 228 Kb), their sequences were highly conserved (Fig. 4). There were only 11 SNPs between the plasmids of *K. variicola* and *K. pneumoniae*, implying horizontal interspecies plasmid transfer according to the empirical threshold (Additional file 1: Fig. S2) [19]. In another *K. variicola* isolate (DSMCPE46 from Case 6), pKPCVIM.1 was integrated into the chromosome; consequently, it was present in all isolates analyzed. No previously reported plasmid was identified with a sequence similar to pKPCVIM.1.

**Fig. 5:**
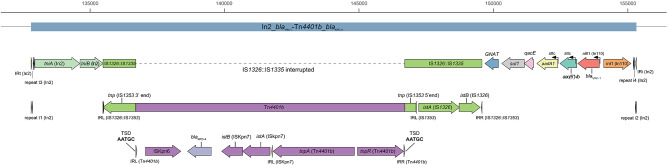
Schematic diagram of the In2_*bla*_VIM−1_-Tn*4401b*_*bla*_KPC−4_ mobile genetic element

On pKPCVIM.1, *bla*_KPC−4_ and *bla*_VIM−1_ were closely positioned, with *bla*_KPC−4_ on Tn*4401b* and *bla*_VIM−1_ located within a gene cassette in the Class 1 integron associated with IS*1326*::IS*1353* (Fig. 5). This integron contained *aac(6’)-Ib*, *aadA1*, *qacEdelta1*, and *sul1*, constituting In110 (*bla*_VIM−1_-*aac(6’)-Ib*-*aadA1*) within a defective Tn*402tni* module (∆*tniB* and *tniA*), truncated by IS1326::IS1353 insertions. This arrangement resembled that of In2 [22, 23]. Notably, a novel 5-bp target site duplication (TSD) was identified in the flaking sequence of the Tn*4401b* (Fig. 5; Additional file 1: Fig. S3) [24, 25]. Additionally, Tn carrying *bla*_KPC−4_ was inserted into IS*1326*::IS*1353* within this integron, forming a novel MGE (In2_*bla*_VIM−1_-Tn*4401b*_*bla*_KPC−4_), which was also present in pKPCVIM.2. Furthermore, pKPCVIM.1 had another Class I integron carrying *bla*_OXA−2_, *aac(6’)-Ib*, *qacEdelta1*, and *sul1* (Additional file 1: Fig. S4). None of the previously reported sequences were identified with > 80% query coverage and identity to In2_*bla*_VIM−1_-Tn4401b_*bla*_KPC−4_.

Although both *K. pneumoniae* and *K. variicola* harbored the plasmid carrying In2-*bla*_VIM−1_-Tn*4401b*_*bla*_KPC−4_ MGE, species-specific differences emerged. Specifically, *K. variicola* isolates harbored additional ARGs (*qnrA1*, *ampR*, *mrx*, *mphA*, and *dfrA12*) and mercury resistance genes (MerEDACPTR) on both pKPCVIM.1 and pKPCVIM.2. Conversely, *K. pneumoniae* isolates contained two additional plasmids: one with *bla*_CTX−M−15_, *bla*_TEM−2_, and *qnrS* genes (pCTXM15) and another 168Kb-sized plasmid of unknown function without antimicrobial resistance genes (Additional file 1: Table S3).

Mapping of the draft genomes of the remaining isolates (DSMCPE40, DSMCPE42, DSMCPE43, and DSMCPE50) to pDSMCPE25.1 as a reference genome revealed the presence of pKPCVIM.1, carrying *bla*_KPC−4_, *bla*_VIM−1_, and *bla*_OXA−2_ in all isolates (Additional file 1: Fig. S5).

## Discussion

This study demonstrated that the hospital-wide spread of *Klebsiella* spp. co-harboring *bla*_KPC−4_ and *bla*_VIM−1_ was facilitated by both clonal transmission and horizontal transfer of the plasmid. WGS revealed that these carbapenemases were integrated into the IncHI5B plasmid as novel MGE constructs (pKPCVIM.1), and the pKPCVIM.1 plasmid spread horizontally between *K. pneumoniae* and *K. variicola*. In this outbreak, clonal transmission of *K. pneumoniae* ST3680 and *K. variicola* ST5252-SLV may have stemmed from healthcare worker contact or movement of patients. Despite an enhanced screening program and infection prevention measures targeting the colonized/infected patients, this strain persisted in a handwashing sink, highlighting the importance of environmental sources in CPE spread and appropriate environmental control measures in preventing the spread of carbapenemase genes within hospital settings.

The pKPCVIM.1 plasmid was classified as IncHI5B. Since its first identification in 2013, IncHI5 has been gradually reported. Typically exceeding 200 Kb, IncHI5 plasmids confer resistance to heavy metals and multiple antibiotics [22]. Although IncHI5 plasmids possess a broad host range, *Klebsiella* spp. are the predominant hosts. Most IncHI5 plasmids were identified in China (83%, 54/65), followed by Japan (9%, 6/65) [26]. South Korea reported only one case carrying *aacA4*, *catB*, *qacED*, and *sul1* ARGs [26]. IncHI5 plasmids appear to be pivotal in the rapid dissemination of carbapenemase genes such as *bla*_IMP_, *bla*_VIM_, or *bla*_NDM−1_ among Enterobacterales [27], with *bla*_NDM−1_ frequently identified across China [26].

The co-existence of *bla*_KPC_ and *bla*_VIM_ in Enterobacterales poses a significant challenge for both clinical treatment and infection control. The effectiveness of new β-lactam/β-lactamase inhibitors is limited against both KPC and VIM carbapenemases, as these agents are effective against KPC-producing strains but not MBL-producing ones [28]. In addition, VIM-1 producing isolates exhibited decreased susceptibility to cefiderocol, with 2- to 4-fold higher minimum inhibitory concentrations (MICs) than in isolates carrying other types of carbapenemases [29]. Although no serious clinical outcomes were observed in this study, colonization by these strains may precedes potential clinical infections, thereby increase the morbidity and mortality rates. Furthermore, the unique genomic features of the strains in our study can facilitate the spread of multiple carbapenemases. In previous reports on the co-harboring of *bla*_KPC−4_ and *bla*_VIM−1_, each carbapenemase gene was often located either on a distinct plasmid or on the chromosome [5, 30–32]. However, in our study, Tn*4401b* carrying *bla*_KPC−4_ was inserted into In2 carrying *bla*_VIM−1_, creating a large novel MGE construct (In2_*bla*_VIM−1_-Tn*4401b*_*bla*_KPC−4_). A novel 5-bp TSD identified in the flanking sequence of Tn*4401* further supports our findings and indicates that Tn*4401* can transpose without target site specificity. This stable construct on the plasmid raises concerns regarding potential diverse ARG recombination and widespread dissemination among Enterobacterales. Its persistence in the environment suggests stable survival within a biofilm formed in the hospital plumbing. Although a definitive source remains elusive, strains carrying this plasmid may have been introduced into the premise plumbing through a patient admitted in 2019. Notably, sink drain biofilms serve as important CPE reservoirs, facilitating plasmid transfer between organisms [33, 34]. Therefore, routine disinfection of sinks and avoiding equipment storage and medication preparation near sinks may reduce transmission risks.

In our study, colistin MICs in isolates with a truncating mutation in *mgrB* ranged from 16 to 128 mg/L, whereas those in isolates without the *mgrB* mutation ranged from 0.5 to 4 mg/L (Table 2). *mgrB* plays an important role in regulating colistin resistance in bacteria, particularly *K. pneumoniae* [35]. Truncating mutation- or insertion sequence-induced *mgrB* inactivation or downregulation has been identified in *K. pneumoniae* isolates [35], underscoring its importance as a common mechanism for colistin resistance development. These findings highlight the significant challenge posed by the reliance on one of the few remaining antibiotics effective against CPE.

The limitations of this study include the lack of isolates from the first and last cases, hindering comprehensive phylogenetic analysis. Environmental cultures conducted over a year post-outbreak further limited the identification of a definitive etiological source. Nevertheless, identifying *K. pneumoniae* strains co-harboring *bla*_KPC−4_ and *bla*_VIM−1_ in patients and a sink long after the outbreak highlights environmental involvement in CPE spread in healthcare settings.

## Conclusion

In conclusion, we identified *Klebsiella* spp. co-harboring *bla*_KPC−4_ and *bla*_VIM−1_ and demonstrated that carbapenemase genes could spread through the horizontal transfer of the novel MGE construct. The persistence of *K. pneumoniae* with this MGE in a hospital sink indicates its potential for persisting in environmental reservoirs. These isolates were also resistant to colistin, which may limit their clinical treatment and public health management. Utilizing WGS to understand the transmission mechanisms allows for the identification of epidemiological links that are difficult to identify through conventional infection control methods. This can further inform the development of targeted interventions and training of healthcare personnel to improve compliance with infection prevention practices.

## Electronic supplementary material

Below is the link to the electronic supplementary material.

Supplementary Material Additional file 1: Supplemental Methods; Fig. S1. Schematic of ward and ICU locations of the hospital; Fig. S2. SNP difference matrix for pKPCVIM.1 detected in *K. pneumoniae* (KP) and *K. variicola* (KV); Fig. S3. Comparison of Tn4401 with previous sequence data; Fig. S4. Schematic diagram of gene structure of Class I integron carrying *bla*_OXA−2_; Fig. S5. Comparison of plasmids carrying *bla*_KPC−4_, *bla*_VIM−1_, and *bla*_OXA−2_ genes; Table S2. Antimicrobial susceptibility profile of *Klebsiella* spp. harboring *bla*_KPC−4_ and *bla*_VIM−1_; Table S3. Characteristics of plasmids harboring *bla*_KPC−4_ and *bla*_VIM−1_ (pKPCVIM.1 and pKPCVIM.2) and *bla*_CTX−M−15_ (pCTXM15)

Supplementary Material Additional file 2: Table S1. Genomic characteristics and antimicrobial resistance genes

## Data Availability

Raw sequencing reads have been deposited in the Sequence Read Archive under accession number PRJNA1079714.
