# Supplementary material for: An outbreak of blaKPC−4- and blaVIM−1-producing Klebsiella pneumoniae and Klebsiella variicola at a single hospital in South Korea
Source: Antimicrob Resist Infect Control. 2024 Oct 11;13:123. doi: 10.1186/s13756-024-01478-2 (PMC11470574; doi:10.1186/s13756-024-01478-2)
Supplement: Supplementary file 1 — Supplementary Material Additional file 1: Supplemental Methods; Fig. S1. Schematic of ward and ICU locations of the hospital; Fig. S2. SNP difference matrix for pKPCVIM.1 detected in K. pneumoniae (KP) and K. variicola (KV); Fig. S3. Comparison of Tn4401 with previous sequence data; Fig. S4. Schematic diagram of gene structure of Class I integron carrying blaOXA−2; Fig. S5. Comparison of plasmids carrying blaKPC−4, blaVIM−1, and blaOXA−2 genes; Table S2. Antimicrobial susceptibility profile of Klebsiella spp. harboring blaKPC−4 and blaVIM−1; Table S3. Characteristics of plasmids harboring blaKPC−4 and blaVIM−1 (pKPCVIM.1 and pKPCVIM.2) and blaCTX−M−15 (pCTXM15) [file 13756_2024_1478_MOESM1_ESM.pdf]

Supplementary Materials for

An outbreak of *bla*$_{KPC-4}$- and *bla*$_{VIM-1}$-producing *Klebsiella pneumoniae* and *Klebsiella variicola* at a single hospital in South Korea

Table of Contents

Supplemental Methods……………………………………………………………page 2

Fig. S1…………………………………………………………………………………page 6

Fig. S2…………………………………………………………………………………page 7

Fig. S3…………………………………………………………………………………page 8

Fig. S4…………………………………………………………………………………page 9

Fig. S5………………………………………………………………………………..page 10

Table S2……………………………………………………………………………page 11

Table S3……………………………………………………………………………page 12

Supplemental References…………………………………………………………page 13

Supplemental Methods

High molecular DNA isolation

High molecular weight DNA was extracted from the nine strains. Bacterial strains were cultured overnight in 10 mL of Todd Hewitt Broth (THB) (Difco™) broth at 37 °C and 5 % CO_2. After the incubation, the cultured medium was centrifuged at 4°C, 4,000 x g for 5 min, and the supernatant was removed. The cell pellet was re-suspended in 300 ul of Tris-EDTA (TE) buffer (10 mM Tris HCl, 1 mM EDTA, pH 8) containing 10 ul of RNase (Sigma) and 20 ul of lysozyme (Sigma), and then the further incubation was performed at 37 °C for 30 min. Following the lysozyme and RNase incubation, 1.7 ml of lysis solution (10 mM Tris HCl, 1 M NaCl, 100 mM EDTA, 0.5% Brij-58, 0.5% sodium lauroylsarcosine, pH 8.0) with 20 ul of proteinase K (Qiagen) was added. The mixture was incubated at 65 °C for 10 min in a water bath. After the lysis step, 2 ml of phenol-chloroform solution (1:1, vol/vol) were added. It was centrifuged at 12,000 x g, 4 °C for 30 min. Upper aqueous phase transferred to a new 1.5 mL centrifuge tube. The aqueous part was further washed with chloroform (1:1, vol/vol), and then it was centrifuged. Following the extraction, it was precipitated with isopropanol and the precipitated pellet was washed with 70 % ethanol. After aspiration the remain ethanol, the pellet was re-solved with the solution of 10 mM Tris-HCl, pH 8.3, and the purified DNA stored at -20 °C until used.

Whole genome sequencing and assembly

Short-read sequencing libraries were prepared for each bacterial DNA sample using the TruSeq Nano DNA sample preparation kit (Illumina, San Diego, CA). Subsequently, the sequencing libraries were pooled and subjected to sequencing on the Illumina NovaSeq 6000 instrument for 300 cycles (2 x 150-bp paired-end). Sequencing adapters and low-quality bases were trimmed using Trimmomatic (version 0.39) [1]. The trimmed reads were assembled using

SPAdes (version 3.15.4) with default parameters [2], and the contigs were further scaffolded using MeDuSa (version 1.6) [3]. Contigs shorter than 200 bp were removed. QUAST (version 5.2.0) was used to generate assembly statistics [4].

For the five strains selected for long-read sequencing, SMRT (single molecule, real-time) sequencing libraries were constructed using the SMRTbell Express Template Prep Kit 2.0 (PacBio, Menlo Park, CA) and sequenced on the RSII (DSMCPE25 and DSMCPE65) or Sequel (DSMCPE46, DSMCPE56, and DSMCPE136) instrument (PacBio). SMRT cell data from the RSII instrument were *de novo* assembled using the RS HGAP assembly (version 3.0) included in SMRT Portal, while those from the Sequel instrument were assembled using the microbial assembly application of SMRT Link based on the HGAP assembly version 4.0. Further corrections were conducted by using Pilon (version 1.21) together with Illumina short-reads to improve assembly quality by correcting bases, fixing mis-assemblies, and filling gaps [5]. Raw sequencing reads have been deposited in the Sequence Read Archive under the accession number PRJNA1079714.

Molecular typing

Kraken2 (version 2.1.3) was used to determine the species of each strain [6]. The identified *Klebsiella* spp. genomes were further confirmed using Kleborate (version 2.3.2) [7]. Multilocus sequence typing (ST) was predicted from the sequences using the Kleborate [7] and allelic profile of the seven housekeeping genes (*gapA*, *infB*, *mdh*, *pgi*, *phoE*, *rpoB*, and *tonB*) [8]. Acquisition of antimicrobial resistance genes, including carbapenemase gene, and their mutations were detected using Kleborate [7] employing the Comprehensive Antibiotic Resistance Database [9]. Capsule (K-) and O- antigen biosynthesis locus typing was performed using Kaptive [10], while the virulence loci and *wzi* alleles were determined using the BIGSdb [8]. The mobilomes were identified using IntegronFinder (version 2.0.2) [11] and

MobileElementFinder (version 1.0.6) [12]. Plasmid sequence comparison was performed using BLAST analysis and Proksee [13]. The gene cassette surrounding *bla*$_{KPC-4}$, *bla*$_{VIM-1}$, and *bla*$_{OXA-2}$ was visualized using Snapgene software (GSL Biotech, San Diego, CA) and gggenes r-package [14]. For strains with only Illumina sequencing data, the presence of MGEs carrying *bla*$_{KPC-4}$ and *bla*$_{VIM-1}$ was confirmed by BLAST, defining a positive hit as a percent identity of ≥ 95% and percent coverage of ≥ 95%. The similarity of the plasmids was assessed by the average nucleotide identity (ANI) between the core genes of the isolates using pANIto (https://github.com/sanger-pathogens/panito) [15]. Details of the genome-based molecular typing are listed in Supplementary Table 1.

Plasmid classification was performed based on replicon and MOB typing, using PlasmidFinder (version 2.1) [16], KpVR (version 1.0) [17], and MOB-Suite (version 3.10) [18]. Plasmid mobility was predicted according to the MOB-Suite (version 3.10.): conjugative, if plasmids contain at least one relaxase and a mate-pair formation marker; 2) mobilizable, if plasmids contain either a relaxase or an oriT but lack the mate-pair formation marker; 3) non-mobilizable, if plasmids lack both relaxase and oriT [18]. Presence of IncHI5 plasmids was further identified using repHI5B on pKOX_R1 (accession number: CP003684) for BLASTN alignment (>95% identity and 100% coverage) with GenBank of NCBI [19].

Phylogenetic analysis

The *de novo* assemblies were annotated using Prokka (version 1.14.6) with default parameters [20]. A core gene alignment for the seven *K. pneumoniae* strains was created using Roary (version 3.13.0) [21]. A maximum likelihood phylogenetic tree based on core gene single nucleotide polymorphisms (SNPs) was constructed using RAxML (version 8.2.12) with 10,000 bootstraps and a generalized time-reversible gamma model [22]. The *K. pneumoniae subsp. pneumoniae* HS11286 (GenBank accession: CP003200) was used as an outgroup to root the

tree. The resulting phylogenetic tree was visualized in Microreact [23]. The chromosomal ANI between isolates was also calculated from the core genes using pANIto [15].

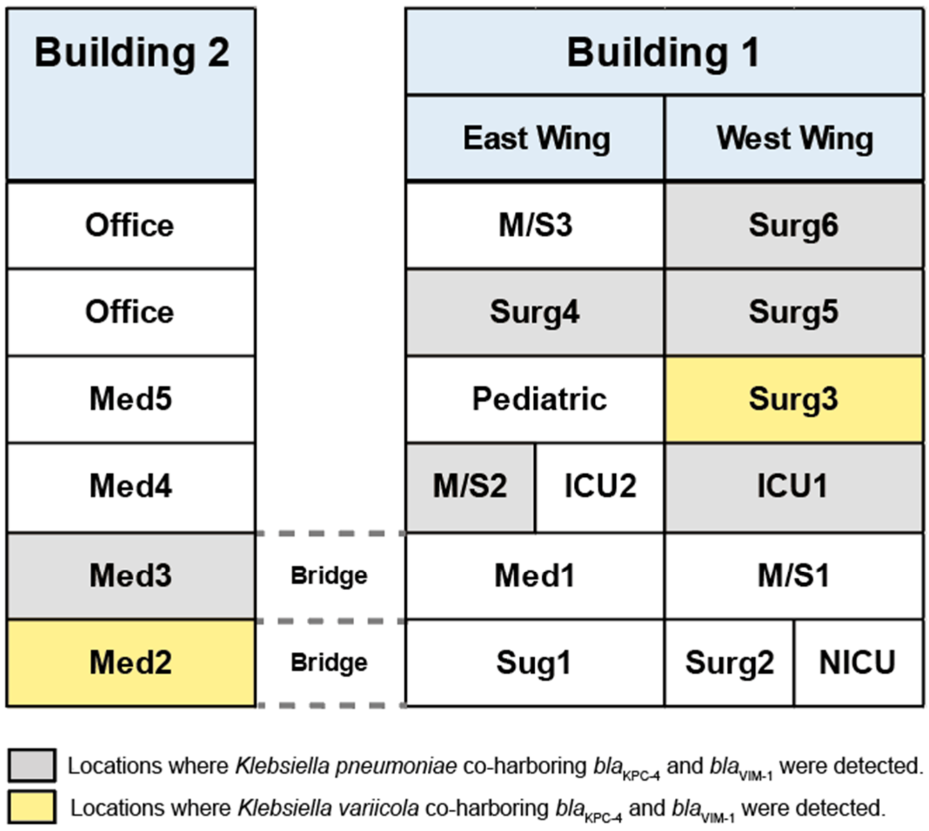

Fig. S1. Schematic of ward and ICU locations of the hospital. MED: medical ward, Surg: surgical ward, M/S: medical and surgical ward, ICU: intensive care unit, NICU: neonatal intensive care unit.

	pDSMCPE56.1 (KP)	pDSMCPE65.1 (KP)	pDSMCPE136.1 (KP)	pDSMCPE25.1 (KV)
pDSMCPE56.1 (KP)	0	0	0	11
pDSMCPE65.1 (KP)	0	0	0	11
pDSMCPE136.1 (KP)	0	0	0	11
pDSMCPE25.1 (KV)	11	11	11	0

Fig. S2. SNP difference matrix of pKPCVIM.1 detected in *K. pneumoniae* (KP) and *K. variicola* (KV).

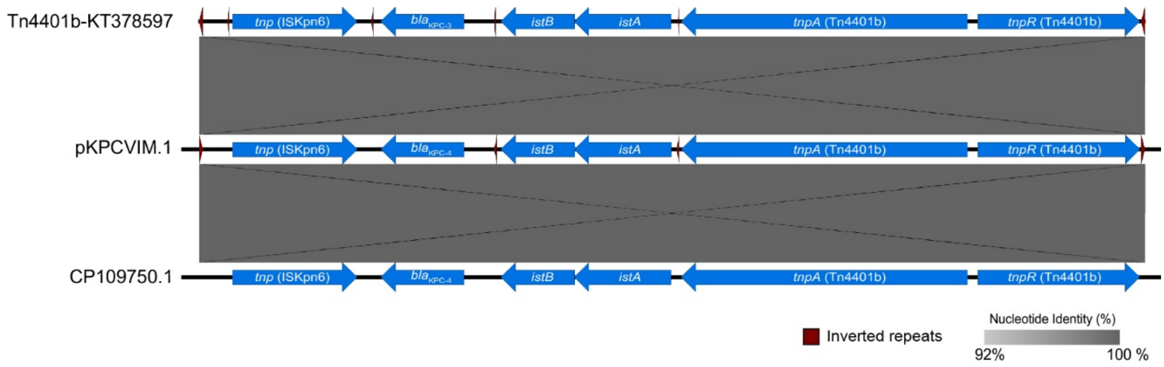

Fig. S3. Comparison of Tn*4401* with previous sequence data. BLAST results against the TnCentral and NCBI nucleotide databases identified Tn4401b (GenBank accession number: KT378597) and *Enterobacter hormaechei* strain 2017-45-136-01-01 plasmid p2017-45-136-01-01_3 (GenBank accession number: CP109750) as the most similar to Tn4401 in pKPCVIM1.1.

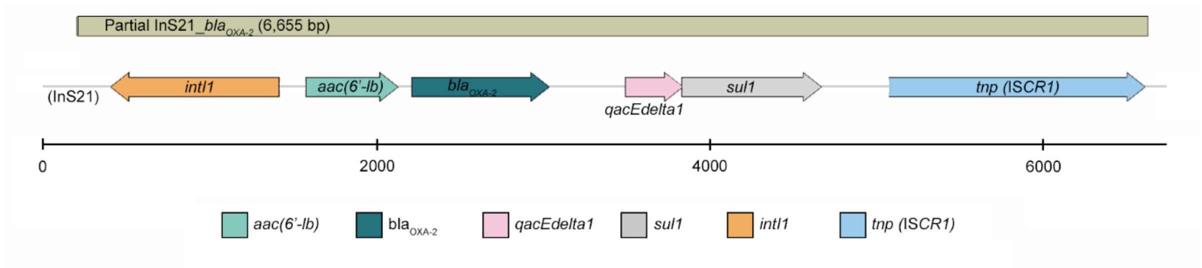

Fig. S4. A schematic diagram of gene structure of Class I integron carrying *bla*OXA-2. pKPCVIM.1 had Class I integron carrying *bla*OXA-2, *aac(6')-Ib*, *qacEdelta1*, and *sul1*, partially identical to InS21_*bla*OXA-2 [24].

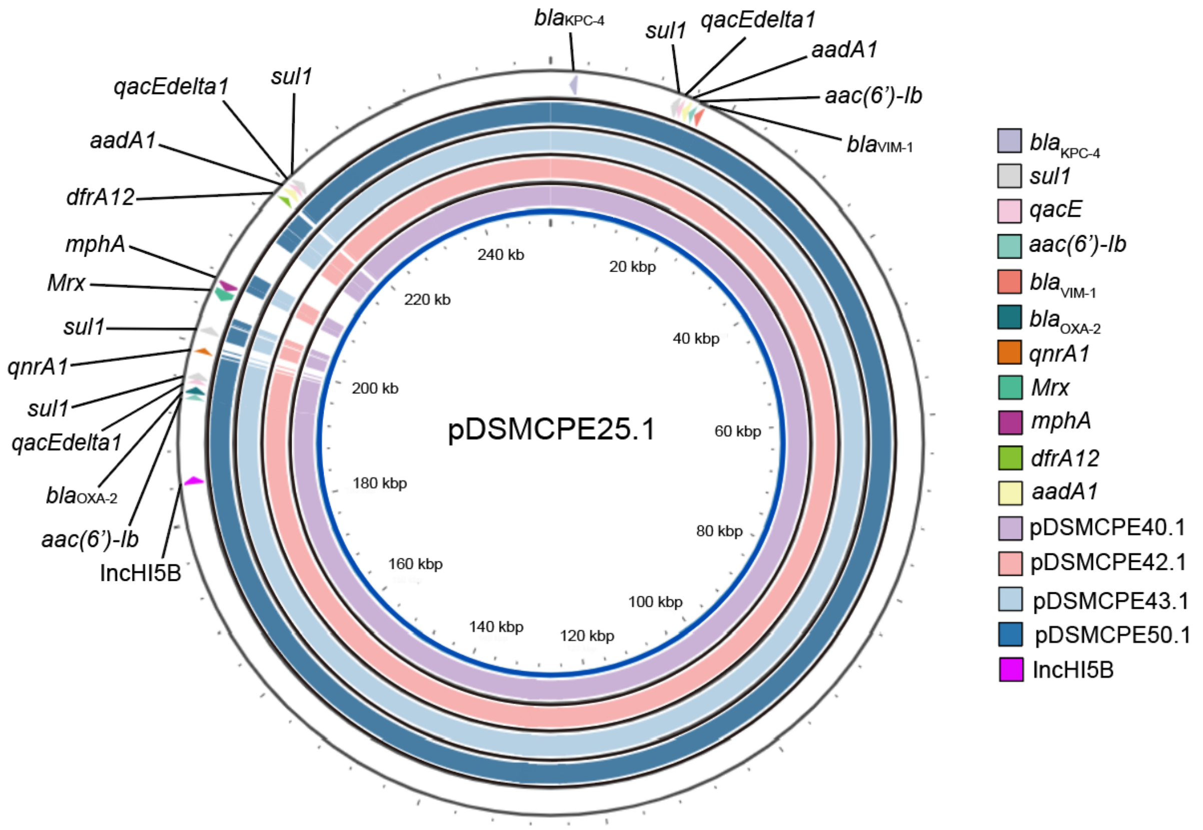

Fig. S5. Comparison of plasmids carrying the *bla*KPC-4, *bla*VIM-1, and *bla*OXA-2 genes. BLAST alignment of four *K. pneumoniae* isolates for which only Illumina sequencing data was produced are shown. The circular map was generated using Proksee, and 252-Kb sized plasmid recovered from the DSMCPE25 (pDSMCPE25.1) was used as the reference sequence.

Table S2. Antimicrobial susceptibility profile of *Klebsiella* spp. harboring $bla_{\text{KPC-4}}$ and $bla_{\text{VIM-1}}$

Isolate ID	Antimicrobial susceptibility test (MIC, µg/mL)																				
	**MER**	**ERT**	**IPM**	**COL**	**GEN**	**AN**	**TOB**	**CIP**	**LVX**	**SXT**	**TET**	**TGC**	**FOS**	**AMP**	**SAM**	**PIP**	**TZP**	**AZT**	**CTX**	**CAZ**	**FEP**
DSMCPE25	64	64	32	2	8	<=16	>8	<=1	<=2	>2/38	<=4	<=1	<=32	>16	>16/8	>64	>64	>16	>32	>16	>16
DSMCPE46	64	32	32	4	8	<=16	>8	<=1	<=2	>2/38	<=4	<=1	<=32	>16	>16/8	>64	>64	>16	>32	>16	>16
DSMCPE40	8	8	16	64	8	<=8	>8	2	2	<=2/38	<=4	<=1	<=32	>16	>16/8	>64	>64	>16	>32	>16	>16
DSMCPE42	32	16	16	32	8	<=8	>8	>2	2	<=2/38	<=4	<=1	<=32	>16	>16/8	>64	>64	>16	>32	>16	>16
DSMCPE43	32	16	16	64	8	<=8	>8	>2	4	<=2/38	<=4	<=1	<=32	>16	>16/8	>64	>64	>16	>32	>16	>16
DSMCPE50	32	16	16	0.5	8	<=8	>8	<=0.5	<=1	<=2/38	<=4	<=1	<=32	>16	>16/8	>64	>64	>16	>32	>16	>16
DSMCPE56	32	16	32	16	8	<=8	>8	>2	2	<=2/38	<=4	<=1	<=32	>16	>16/8	>64	>64	>16	>32	>16	>16
DSMCPE65	64	32	32	32	8	<=8	>8	>2	4	<=2/38	<=4	<=1	<=32	>16	>16/8	>64	>64	>16	>32	>16	>16
DSMCPE136	64	32	64	128	8	<=8	>8	>2	4	<=2/38	<=4	<=1	<=32	>16	>16/8	64	>64	>16	>32	>16	>16

MIC: minimum inhibitory concentration, MER: meropenem, ERT: ertapenem, IPM: imipenem, COL: colistin, GEN: gentamicin, AN: amikacin, TOB: tobramycin, CIP: ciprofloxacin, LVX: levofloxacin, SXT: trimethoprim/sulfamethoxazole, TET: tetracycline, TGC: tigecycline, FOS: fosfomycin, AMP: ampicillin, SAM: ampicillin/sulbactam, PIP: piperacillin, TZP: piperacillin/tazobactam, AZT: aztreonam, CTX: cefotaxime, CAZ: ceftazidime, FEP: cefepime.

Table S3. Characteristics of plasmid characteristics harboring bla_{KPC-4} and bla_{VIM-1} (pKPCVIM.1 and pKPCVIM.2) and $bla_{CTX-M-15}$ (pCTXM15).

Plasmid type	Replicase	Relaxase	Mobility	Plasmids of Isolates	Size (bp)	Antimicrobial Resistance Genes			
						Beta-lactam	Aminoglycoside	Quinolone	Other antibiotics
pKPCVIM.1	IncHI5B	MOBH	Conjugative	pDSMCPE25.1	251,794	bla_{KPC-4}, bla_{VIM-1}, bla_{OXA-2}	*aac(6')-Ib8, aac(6')-Ib9, ant(3")-Ia, ant(3")-Ia*	*qnrA1*	*qacEdelta1, sul1, qacEdelta1, sul1, mrx, mphA, dfrA12*
				pDSMCPE56.1	226,907	bla_{KPC-4}, bla_{VIM-1}, bla_{OXA-2}	*aac(6')-Ib8, aac(6')-Ib9, ant(3")-Ia*		*qacEdelta1, sul1, qacEdelta1, sul1*
				pDSMCPE65.1	227,264	bla_{KPC-4}, bla_{VIM-1}, bla_{OXA-2}	*aac(6')-Ib8, aac(6')-Ib9, ant(3")-Ia*		*qacEdelta1, sul1, qacEdelta1, sul1*
				pDSMCPE136.1	228,870	bla_{KPC-4}, bla_{VIM-1}, bla_{OXA-2}	*aac(6')-Ib8, aac(6')-Ib9, ant(3")-Ia*		*qacEdelta1, sul1, qacEdelta1, sul1*
pKPCVIM.2	IncR	MOBF	Non-mobilizable	pDSMCPE25.2	145,583	bla_{KPC-4}, bla_{VIM-1}	*aac(6')-Ib9, ant(3")-Ia*		*qacEdelta1, sul1*
				pDSMCPE46.2	145,582	bla_{KPC-4}, bla_{VIM-1}	*aac(6')-Ib9, ant(3")-Ia*		*qacEdelta1, sul1*
pCTXM15	IncFII(K), IncR	MOBF	Mobilizable	pDSMCPE56.3	91,678	$bla_{CTX-M-15}$, bla_{TEM-1}		*qnrS1*	
				pDSMCPE65.3	94,729	$bla_{CTX-M-15}$, $bla_{CTX-M-15}$, bla_{TEM-1}		*qnrS1*	
				pDSMCPE136.3	94,729	$bla_{CTX-M-15}$, $bla_{CTX-M-15}$, bla_{TEM-1}		*qnrS1*	
Other plasmids	IncFIB	-	Non-mobilizable	pDSMCPE56.3	168,415				
				pDSMCPE65.3	168,415				
				pDSMCPE136.3	168,415				

Supplemental References

1. Bolger AM, Lohse M, Usadel B. Trimmomatic: a flexible trimmer for Illumina sequence data. Bioinformatics **2014**; 30(15): 2114-20.
2. Prjibelski A, Antipov D, Meleshko D, Lapidus A, Korobeynikov A. Using SPAdes De Novo Assembler. Curr Protoc Bioinformatics **2020**; 70(1): e102.
3. Bosi E, Donati B, Galardini M, et al. MeDuSa: a multi-draft based scaffolder. Bioinformatics **2015**; 31(15): 2443-51.
4. Gurevich A, Saveliev V, Vyahhi N, Tesler G. QUAST: quality assessment tool for genome assemblies. Bioinformatics **2013**; 29(8): 1072-5.
5. Walker BJ, Abeel T, Shea T, et al. Pilon: an integrated tool for comprehensive microbial variant detection and genome assembly improvement. PLoS One **2014**; 9(11): e112963.
6. Lu J, Rincon N, Wood DE, et al. Metagenome analysis using the Kraken software suite. Nat Protoc **2022**; 17(12): 2815-39.
7. Lam MMC, Wick RR, Watts SC, Cerdeira LT, Wyres KL, Holt KE. A genomic surveillance framework and genotyping tool for Klebsiella pneumoniae and its related species complex. Nat Commun **2021**; 12(1): 4188.
8. Jolley KA, Maiden MC. BIGSdb: Scalable analysis of bacterial genome variation at the population level. BMC Bioinformatics **2010**; 11: 595.
9. Alcock BP, Raphenya AR, Lau TTY, et al. CARD 2020: antibiotic resistome surveillance with the comprehensive antibiotic resistance database. Nucleic Acids Res **2020**; 48(D1): D517-D25.
10. Lam MMC, Wick RR, Judd LM, Holt KE, Wyres KL. Kaptive 2.0: updated capsule and lipopolysaccharide locus typing for the Klebsiella pneumoniae species complex. Microb Genom **2022**; 8(3).
11. Neron B, Littner E, Haudiquet M, Perrin A, Cury J, Rocha EPC. IntegronFinder 2.0: Identification and Analysis of Integrons across Bacteria, with a Focus on Antibiotic Resistance in Klebsiella. Microorganisms **2022**; 10(4).
12. Johansson MHK, Bortolaia V, Tansirichaiya S, Aarestrup FM, Roberts AP, Petersen TN. Detection of mobile genetic elements associated with antibiotic resistance in Salmonella enterica using a newly developed web tool: MobileElementFinder. J Antimicrob Chemother **2021**; 76(1): 101-9.
13. Grant JR, Enns E, Marinier E, et al. Proksee: in-depth characterization and visualization of bacterial genomes. Nucleic Acids Res **2023**; 51(W1): W484-W92.
14. Wilkins D. gggenes: Draw Gene Arrow Maps in 'ggplot2'_.R package version 0.4.1, <https://CRAN.R-project.org/package=gggenes>. **2020**.
15. David S, Reuter S, Harris SR, et al. Epidemic of carbapenem-resistant Klebsiella pneumoniae in Europe is driven by nosocomial spread. Nat Microbiol **2019**; 4(11): 1919-29.
16. Carattoli A, Zankari E, Garcia-Fernandez A, et al. In silico detection and typing of plasmids using PlasmidFinder and plasmid multilocus sequence typing. Antimicrob Agents Chemother **2014**; 58(7): 3895-903.
17. Tian D, Wang M, Zhou Y, Hu D, Ou HY, Jiang X. Genetic diversity and evolution of the virulence plasmids encoding aerobactin and salmochelin in Klebsiella pneumoniae. Virulence **2021**; 12(1): 1323-33.
18. Robertson J, Nash JHE. MOB-suite: software tools for clustering, reconstruction and typing of plasmids from draft assemblies. Microb Genom **2018**; 4(8).
19. Sayers EW, Beck J, Bolton EE, et al. Database resources of the National Center for Biotechnology Information. Nucleic Acids Res **2021**; 49(D1): D10-d7.
20. Seemann T. Prokka: rapid prokaryotic genome annotation. Bioinformatics **2014**; 30(14):

2068-9.
21. Page AJ, Cummins CA, Hunt M, et al. Roary: rapid large-scale prokaryote pan genome analysis. Bioinformatics **2015**; 31(22): 3691-3.
22. Stamatakis A. RAxML version 8: a tool for phylogenetic analysis and post-analysis of large phylogenies. Bioinformatics **2014**; 30(9): 1312-3.
23. Argimon S, Abudahab K, Goater RJE, et al. Microreact: visualizing and sharing data for genomic epidemiology and phylogeography. Microb Genom **2016**; 2(11): e000093.
24. Di Conza J, Ayala JA, Power P, Mollerach M, Gutkind G. Novel class 1 integron (InS21) carrying blaCTX-M-2 in Salmonella enterica serovar infantis. Antimicrob Agents Chemother **2002**; 46(7): 2257-61.
